# From pharmacokinetics to precision dosing: optimizing continuous infusion regimens of ciprofol for elderly patients

**DOI:** 10.3389/fphar.2026.1764590

**Published:** 2026-02-02

**Authors:** Jiaxi Zhu, Jing He, Bowen Zhong, Ying Cao, Xingan Zhang, Bo Xu

**Affiliations:** 1 Department of Anesthesiology, General Hospital of Southern Theater Command of PLA, Guangzhou, Guangdong, China; 2 The First School of Clinical Medicine, Southern Medical University, Guangzhou, Guangdong, China

**Keywords:** ciprofol, elderly patients, pharmacodynamics, pharmacokinetics, three-compartment model

## Abstract

**Objective:**

To develop and validate a population pharmacokinetic/pharmacodynamic (PK/PD) model for ciprofol in elderly surgical patients, delineating its pharmacokinetic profile and concentration-effect relationship to inform precision dosing.

**Methods:**

Twenty patients (aged ≥65 years) undergoing elective surgery were enrolled. We performed population PK/PD analysis using nonlinear mixed-effects modeling on 386 arterial blood samples and synchronized Bispectral Index (BIS) data. A linear three-compartment model and a sigmoid Emax model described the PK and PD (BIS), respectively. Covariates (age, weight, gender, and laboratory parameters) were tested via stepwise selection. Model performance was evaluated using goodness-of-fit plots, bootstrap (*n* = 1,000), and prediction-corrected visual predictive checks. Dosing regimens were optimized via Monte Carlo simulation.

**Results:**

A three-compartment model best described the PK. The center volume (V_1_) was generally approximated at 2.95 L, but the peripheral volumes (V_2_ and V_3_) were 45.15 L and 76.79 L, respectively. The clearance (CL) was assessed at 1.01 L min^-1^. Body weight and age significantly influenced CL. PD analysis showed rapid effect-site equilibration (K_e0_: 1.09 min^-1^), with an EC_50_ of 233.91 ng mL^-1^ and a Hill coefficient of 3.00. No covariates significantly affected PD parameters. The model exhibited sufficient fit and strong predictive efficacy. The simulation results confirmed that administering an intravenous loading dose of 0.4 mg kg^-1^ over 1 min, followed by an initial continuous infusion at a rate of 0.6 mg kg^-1^·h^-1^ for 2 h, could stably maintain the patients’ BIS values within the target range of 40–60.

**Conclusion:**

A population PK/PD model for ciprofol in elderly patients was successfully established and validated. The model supports optimized, individualized dosing to achieve target anesthesia depth in this population.

## 1 Introduction

With the intensification of the global aging trend, the demand for surgery and intensive care among elderly patients has been continuously increasing, making anesthesia and sedation management a crucial challenge for perioperative safety. Due to the decline in organ functions (e.g., decreased glomerular filtration rate, reduced activity of hepatic metabolic enzymes, and increased body fat ratio) and the presence of multiple comorbidities in the elderly population, the pharmacokinetic (PK) and pharmacodynamic (PD) profiles of anesthetic drugs in this cohort have undergone significant alterations (Coetzee and Absalom, 2025; Ngcobo, 2025). Traditional dosing regimens are prone to causing adverse events such as excessive sedation, hemodynamic instability, or delayed emergence, which in turn elevate the risk of postoperative complications including myocardial injury and neurocognitive disorders (Happ et al., 2025; Strøm et al., 2016). Therefore, optimizing anesthetic dosing strategies for elderly patients to achieve individualized precision medicine has become a key research topic in current anesthesiology.

Ciprofol is a novel intravenous anesthetic independently developed in China. Through stereoselective modification of the chemical structure of propofol, it has significantly enhanced affinity for γ-aminobutyric acid type A (GABAA) receptors (Chen et al., 2022; Ding et al., 2022). This drug is characterized by rapid onset of action, fast recovery, and a low incidence of injection pain. Preliminary clinical trials in adult populations have demonstrated that compared with conventional propofol, ciprofol exhibits superior performance in maintaining hemodynamic stability and reducing the risk of respiratory depression (Gao et al., 2024; Liang et al., 2023; Liang et al., 2024; Wang et al., 2022). Ciprofol is mainly metabolized via oxidation and glucuronidation pathways, with metabolites excreted through urine, laying a solid foundation for good safety. These pharmacological properties render it promising for application in elderly patients, who typically have diminished physiological reserve capacity. For instance, a clinical trial indicated that in the anesthesia of elderly patients undergoing endoscopic retrograde cholangiopancreatography (ERCP), the overall incidence of adverse reactions to ciprofol was significantly lower than that in the propofol group, with patients also experiencing faster recovery (Ding et al., 2024).

Population pharmacokinetic-pharmacodynamic (PopPK/PD) modeling is an essential method for clarifying the concentration-effect relationship of drugs within particular populations. It enables quantification of intra-individual and inter-individual variability, as well as identification of key covariates affecting drug disposition (Li et al., 2024; Vandemoortele et al., 2022). Dose optimization based on PK/PD models helps improve the safety and efficacy of anesthesia in elderly patients, facilitating individualized drug administration. However, the clinical application of ciprofol in the elderly population currently lacks sufficient pharmacokinetic evidence. Although existing studies have suggested that age, body weight, and gender are important covariates influencing the PK parameters of ciprofol (Liu et al., 2024), most of the established PK/PD models are constructed based on data from the general adult population and have not been fully validated in the elderly subgroup. We need to confirm the predictive accuracy of these models, especially in elderly patients with complex conditions like comorbid renal insufficiency or frailty. Therefore, it is necessary to establish a PopPK/PD model of ciprofol specifically for the elderly population, to clarify the impacts of key covariates including age, renal function, and body composition, and to quantitatively characterize the dose-effect relationship between drug concentration and pharmacodynamic indicators such as the bispectral index (BIS).

This study aims to systematically investigate the pharmacokinetic and pharmacodynamic characteristics of intravenously infused ciprofol in elderly patients through a prospective clinical trial, construct its PopPK/PD model, and identify the key factors affecting drug disposition, thereby providing a scientific basis for the rational clinical application of ciprofol in elderly patients.

## 2 Materials and methods

### 2.1 Study subjects and design

This PK/PD analysis included data from 20 elderly patients who underwent elective surgery from December 2024 to May 2025. The study was conducted after obtaining approval from the Ethics Committee of the General Hospital of Southern Theater Command of PLA (No. NZLLKZ2024149) and was prospectively registered at the Chinese Clinical Trial Registry (No. ChiCTR2400093796). All participants provided written informed consent prior to enrollment.

Exclusion criteria included: patients with severe cardiac, pulmonary, hepatic, or renal dysfunction, or coagulation disorders; those with a history of mental illness (e.g., dementia, schizophrenia), long-term use of psychotropic drugs or chronic analgesics, or a history of alcoholism; participants who had been involved in other drug clinical trials within the past 6 months; patients with a known allergy to the study drugs; and those with an expected intraoperative blood loss exceeding 800 mL.

All patients fasted from midnight on the day of surgery and did not receive any preoperatively prescribed medications. Upon entering the operating room, the following monitoring devices were connected: pulse oximeter, electrocardiograph, end-tidal carbon dioxide monitor, non-invasive blood pressure monitor, and bispectral index (BIS®) monitor (Covidien, Boulder, CO, United States). All monitoring data were continuously collected from the start of anesthesia induction until the termination of anesthesia.

The anesthesia implementation and administration regimen in this study strictly followed a standardized clinical pathway, and was designed based on the labeling information of ciprofol approved in China as well as the recommended usage for adult surgical patients reported in relevant clinical research literature (Liang et al., 2023). A 20G arterial catheter was placed in the patient’s radial artery to facilitate repeated arterial blood sampling. Prior to anesthesia induction, preoxygenation was performed by inhaling 100% oxygen via a face mask. Anesthesia induction was achieved with an intravenous bolus of ciprofol 0.4 mg kg^-1^, followed by a continuous infusion of ciprofol at a rate of 0.8 mg kg^-1^·h^-1^ 5 min later until the end of surgery. Meanwhile, remifentanil was administered via target-controlled infusion (TCI) of the effect site using the Minto model (Abad-Torrent et al., 2022).

During anesthesia maintenance, midazolam was intravenously administered as appropriate if the BIS value exceeded 60. The target effect-site concentration (Ce) of remifentanil was titrated according to the patient’s hemodynamic responses, maintained within the range of 2–20 ng ml^-1^ to ensure hemodynamic stability. Cisatracurium besylate 2 mg kg^-1^ was intravenously injected to assist with tracheal intubation. Intraoperative mechanical ventilation was initiated after intubation. The ventilator settings were titrated to maintain an end-tidal partial pressure of carbon dioxide (P_ET_CO_2_) within the target range of 35–45 mmHg for the duration of the procedure. If hemodynamic fluctuations occurred during surgery, vasoactive drugs were used for intervention as needed. The infusion of ciprofol and remifentanil was stopped when the surgical skin suturing began; neostigmine was intravenously injected at the end of surgery to reverse the neuromuscular blocking effect.

### 2.2 Arterial blood sample collection and analysis

Blood samples (2 mL) were obtained via arterial puncture at scheduled times relative to ciprofol administration: at 0, 1, 3, and 5 min after a 1-min bolus injection; at 5, 10, 15, 30, 45, and 60 min after initiating continuous infusion; and at 3, 5, 10, 30 min, 1, 1.5, 2, 4, and 6 h after infusion cessation. Samples were immediately transferred to EDTA tubes, centrifuged (3,000×g, 10 min), and the resulting plasma was stored at −80 °C pending analysis.

Ciprofol plasma concentrations were quantified using a validated UPLC-MS/MS method (Zhu et al., 2025). Following protein precipitation with methanol using ciprofol-d6 as the internal standard, chromatographic separation was achieved on a Shimadzu Shim-pack GIST-HP C18 column (3 μm, 2.1 × 150 mm) maintained at 40 °C. The mobile phase, comprising 5 mmol L^-1^ ammonium acetate (A) and methanol (B), was delivered isocratically at 0.4 mL min^-1^. Detection employed electrospray ionization in negative mode with MRM. The monitored transitions were m/z 203.100 → 175.000 for ciprofol and m/z 209.100 → 181.100 for the internal standard. The method was linear from 5 to 5,000 ng mL^-1^, with an LLOQ of 5 ng mL^-1^.

### 2.3 Construction of population PK model

Population pharmacokinetic nonlinear mixed-effects modeling (PopPK-NLME) was performed using NONMEM 7.2.0 software (ICON Development Solutions, Ellicott City, MD, United States) combined with the gfortran 9.0 compiler. During the modeling process, the Wings for NONMEM (WFN720; http://wfn.sourceforge.net) visualization platform was used to call NONMEM for calculations. The parameter estimation method adopted was First-Order Conditional Estimation with Interaction (FOCEI), which can improve the parameter estimation accuracy of data with significant individual differences by introducing interaction terms between inter-individual variability and fixed effects, making it particularly suitable for heterogeneous study populations such as the elderly.

**FIGURE 1:**
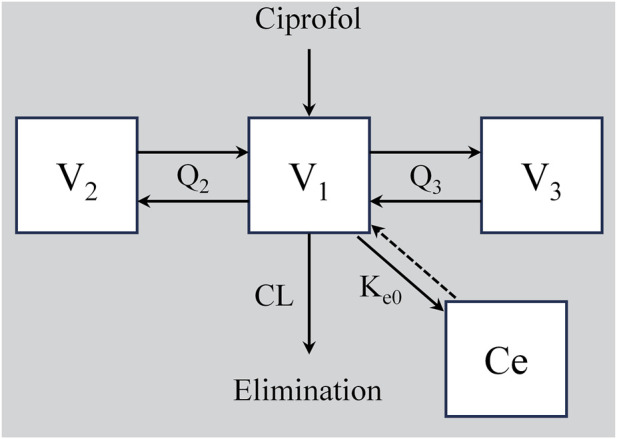
Schematic diagram of the population pharmacokinetic/pharmacodynamic (PK/PD) structural model of ciprofol. V_1_: Volume of distribution in the central compartment; V_2_, V_3_: Volumes of distribution in the peripheral compartments; Q_2_, Q_3_: Inter-compartmental clearances; CL: Drug elimination rate constant of the central compartment; K_e0_: Equilibrium rate constant between the central pharmacokinetic compartment (V_1_) and the central effect compartment. C_e_: Drug concentration in the effect compartment.

The construction of the base model was performed by comparing the one-compartment, two-compartment, and three-compartment structures, which were screened based on the minimization of the objective function value (OFV), parameter precision, and goodness of fit. Statistical analysis showed that the three-compartment model could more accurately characterize the distribution and elimination profiles of ciprofol in elderly patients (Supplementary Material Table S1). These findings were consistent with those of previous studies (Hu et al., 2021; Liu et al., 2024; Teng et al., 2021), and the final model structure is presented in Figure 1.

#### 2.3.1 Construction of the PK model

The inter-individual variability (IIV) model was described using an exponential model to characterize the individual differences in PK parameters, ensuring the non-negativity of parameters:

$$\theta_{i}=\theta_{\text{pop}}\cdot e^{\eta_{i}}$$

where $\theta_{i}$ represents the individual parameter of the i -th patient; $\theta_{\text{pop}}$ denotes the population parameter; $\eta_{i}$ is the inter-individual random error, which follows a normal distribution $N\left(0,\omega^{2}\right)$ ; and ω is the standard deviation of the inter-individual variability model.

The residual variability (RV) model was employed to quantify the deviation between the observed plasma drug concentration values and the predicted values. In this study, we evaluated the proportional residual model, additive residual model, and combined residual model sequentially to identify the optimal approach for characterizing random errors. Model selection was primarily based on changes in the objective function value (OFV) and the uniformity of conditional weighted residuals (CWRES) distribution against predicted concentrations. Comparative results demonstrated that the proportional model yielded the lowest OFV during the fitting process and was significantly superior to the additive and combined models (Supplementary Material Table S2). Therefore, the proportional residual model was ultimately selected, and its calculation formula is presented as follows:

$$C_{\text{obs},i,j}=C_{\text{pred},i,j}\cdot \left(1+\varepsilon_{,i,j}\right)$$

where $C_{\text{obs},i,j}$ and $C_{\text{pred},i,j}$ are the observed and predicted concentrations of the j -th patient at the -th time point, respectively; $\varepsilon_{i,j}$ is the proportional error, following a normal distribution $N\left(0,\sigma^{2}\right)$ .

The OFV of the model serves as an indicator of goodness-of-fit, and its variance conforms to a chi-squared (χ^2^) distribution with approximate degrees of freedom (df). For covariate screening, a forward stepwise univariate analysis was employed, where covariates resulting in an OFV reduction of at least 3.84 (α = 0.05, df = 1) were retained. A backward elimination analysis was subsequently performed for covariates causing an OFV increase of at least 10.82 (α = 0.001, df = 1). Meanwhile, the reduction trends of IIV and RV values were also evaluated.

#### 2.3.2 Construction of the population PD model

An effect-site compartment model was adopted to address the hysteresis between plasma drug concentration and BIS values. The differential equation describing the change in effect-site concentration ( $C_{e}$ ) is expressed as:

$$\frac{dC_{e}}{dt}=k_{e0}\cdot C_{1}\left(t\right)-k_{e0}\cdot C_{e}\left(t\right)$$

where $k_{e0}$ is the transfer rate constant between the effect-site and central compartments. A sigmoidal Emax model was utilized to characterize the concentration-effect relationship between $C_{e}$ and BIS values, with the formula:

$$E\left(t\right)=E_{0}-\frac{E\max*Ce^{\wedge }\gamma }{\left(EC50^{\gamma }+Ce^{\wedge }\gamma \right)}$$

where $E\left(t\right)$ is the BIS value at time; $E_{0}$ represents the baseline BIS value (before drug administration); Emax denotes the maximum BIS suppression effect; $EC_{50}$ is the effect-site concentration required to produce 50% of the maximum effect; and γ (Hill coefficient) is a shape parameter describing the steepness of the concentration-effect curve.

### 2.4 Model evaluation and validation

The final model was assessed for accuracy and stability using diagnostic plots, bootstrap validation, and prediction-corrected visual predictive checks (pc-VPC) (Nguyen et al., 2017). Diagnostic evaluation included goodness-of-fit plots: observations (DV) versus population predictions (PRED) and individual predictions (IPRED), along with CWRES plotted against both PRED and time after dose (TAD). For bootstrap validation, 1,000 replicate datasets were generated by random sampling with replacement from the original data, and model parameters were re-estimated for each. The median parameter estimates from bootstrap replicates aligned closely with the original estimates, all of which fell within the 2.5th–97.5th percentile intervals, indicating robust model stability. Subsequently, pc-VPC was performed by simulating multiple datasets based on the final model. The 5th, 50th, and 95th percentiles of the simulated data at each time point were calculated to construct prediction intervals. Excellent predictive performance was confirmed by the close agreement between the percentiles of the observed data and the simulated prediction intervals across all time points.

### 2.5 Simulation

To predict the target effects of anesthesia induction and maintenance, and provide a basis for dose optimization in future studies, Monte Carlo simulations were performed based on the final validated PK/PD model to estimate the plasma and effect-site concentrations of ciprofol under different dosing regimens. The simulation protocol was designed as follows: a loading dose of 0.4 mg kg^-1^ was administered via intravenous infusion over 1 min, followed by continuous infusion of maintenance doses at 0.4, 0.6, 0.8, 1.0, 1.2, and 1.4 mg kg^-1^·h^-1^, respectively, with a total infusion duration of 2 h. Each simulation included 1,000 virtual subjects, whose PK/PD parameters and inter-individual variability were derived from random sampling of the normal or log-normal distributions of the final model parameters. Through comprehensive comparison of key efficacy indicators across different dosing regimens, the optimal administration strategy that best matched the target effects was finally identified.

## 3 Results

### 3.1 Demographics

**FIGURE 2:**
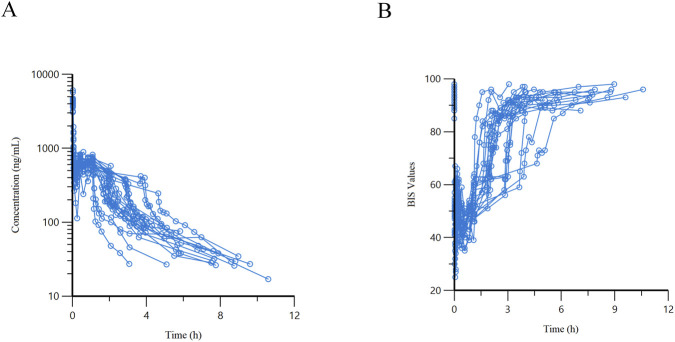
Time-course curves of ciprofol plasma concentration and bispectral index (BIS). Time zero represents the initiation time of ciprofol infusion. **(A)** Observed plasma concentration-time curves of ciprofol in 20 subjects, with scatter points indicating the measured concentration values at each time point. **(B)** BIS—time curves recorded synchronously at blood sampling points in the same cohort of 20 patients.

A total of 20 elderly surgical patients (11 males and 9 females, aged 67–82 years, weighing 49–80 kg) were finally enrolled in this study. A total of 386 arterial plasma samples were collected, and 386 corresponding BIS values were recorded synchronously. Except for the pre-administration time points, the plasma concentrations of ciprofol in all samples were above the LLOQ, and no outliers were identified. The demographic characteristics, laboratory test results, and surgical types of the patients are detailed in Table 1; the medications administered during anesthesia maintenance are presented in Supplementary Material Table S3; and the time-dependent trends of ciprofol plasma concentrations and BIS values are illustrated in Figure 2.

**TABLE 1:** Demographic and clinical characteristics of enrolled patients.

Characteristic	Patients (*n* = 20)
Age (yr)	72.95 ± 4.47 (72.5; 67–82)
Sex (Male/Female) (*n*, %)	11 (55%)/9 (45%)
ASA physcial status II/III (*n*, %)	15 (75%)/5 (25%)
Height (cm)	162.65 ± 6.3 (164; 151–172)
Weight (kg)	65.63 ± 9.34 (66; 49–80)
BMI (kg·m^-2^)	24.7 ± 2.31 (24.91; 21.20–29.38)
BSA (m^2^)	1.72 ± 0.2 (1.71; 1.4–1.94)
IBW (kg)	57.59 ± 6.47 (56.74; 47.18–67.83)
LBM (kg)	48.83 ± 6.79 (47.03; 36.82–61.12)
Creatinine (μmol·L^-1^)	64.65 ± 18.43 (59; 37–107)
GFR (mL·min^-1^·1.73 m^-2^)	79.22 ± 11.23 (80.15; 56.4–98.3)
Albumin (g·L^-1^)	40.4 ± 3.02 (40.15; 36.7–44.7)
Hemoglobin (g·L^-1^)	127.7 ± 14.77 (128; 93–157)
Total protein (g·L^-1^)	69.30 ± 4.6 (69; 61.6–81.6)
Total bilirubin (μmol·L^-1^)	11.38 ± 5.7 (9.35; 5–28.3)
Prothrombin time (s)	13.76 ± 0.53 (13.8; 12.9–14.9)
Alanine aminotransferase (U·L^-1^)	23.85 ± 13.62 (21.5; 8–73)
Aspartate aminotransferase (U·L^-1^)	21.25 ± 3.57 (21.5; 16–28)
Alkaline phosphatase (U·L^-1^)	78.6 ± 18.55 (77; 54–120)
Type of surgery (*n*, %)	​
Orthopedic surgery	8 (40%)
General surgery	6 (30%)
Urological surgery	3 (15%)
Thoracic surgery	3 (15%)

Data are presented as counts or means mean ± standard deviation (median, range) as appropriate. ASA, american society of anesthesiologists; BSA, body surface area; IBW, ideal body weight; LBM, lean body mass; GFR, glomerular filtration rate.

### 3.2 Population PK model

A PopPK model describing the continuous infusion of ciprofol in elderly patients (aged ≥65 years) was successfully established, and the typical parameter estimates are summarized in Table 2. Pharmacokinetic characteristic analysis revealed that ciprofol exhibited rapid distribution and extensive tissue distribution in the elderly population. The model-estimated central compartment volume of distribution (V_1_) was 2.95 L, while the volumes of distribution of the shallow peripheral compartment (V_2_) and deep peripheral compartment (V_3_) were 45.15 L and 76.79 L, respectively, indicating significant drug accumulation in deep tissues. The typical value of systemic clearance (CL) was 1.01 L min^-1^. The inter-compartmental clearances Q_2_ and Q_3_ were 0.76 L min^-1^ and 0.66 L min^-1^, respectively.

**TABLE 2:** Final PK model parameter estimation of ciprofol.

Parameters	Base model	Final model	Bootstrap	95% CI	η-shrinkage (%)	ε-shrinkage (%)
	Estimate (RSE%)	Estimate (RSE%)	Median			
Fixed effects						
V_1_ (L)	2.78 (16.91)	2.95 (8.54)	2.88	2.82, 2.93	22.32	—
V_2_ (L)	42.80 (6.88)	45.15 (6.20)	43.15	39.49, 47.04	25.71	—
V_3_ (L)	75.26 (2.43)	76.79 (5.15)	73.71	70.99, 85.27	21.15	—
CL (L·min^-1^)	0.98 (1.28)	1.01 (7.23)	1.02	0.96, 1.07	24.52	—
Age on CL	—	−0.21 (8.63)	−0.22	−0.20, −0.25	—	—
WT on CL	—	0.74 (12.42)	0.76	0.73, 0.84	—	—
Q_2_ (L·min^-1^)	0.73 (9.92)	0.76 (6.83)	0.77	0.72, 0.82	28.94	—
Q_3_ (L·min^-1^)	0.64 (4.47)	0.66 (3.84)	0.65	0.61, 0.69	23.87	—
Random effects (BSV)						
ω(V_1_)	0.06 (37.16)	0.06 (22.18)	0.06	0.04, 0.08	—	—
ω(V_2_)	0.14 (8.55)	0.11 (9.12)	0.11	0.10, 0.12	—	—
ω(V_3_)	0.12 (12.04)	0.11 (10.36)	0.10	0.08, 0.15	—	—
ω(CL)	0.04 (17.46)	0.03 (18.58)	0.03	0.01, 0.05	—	—
ω(Q_2_)	0.04 (40.09)	0.03 (21.64)	0.03	0.01, 0.07	—	—
ω(Q_3_)	0.02 (12.71)	0.02 (8.75)	0.02	0.01, 0.05	—	—
Random effects (WSV)						
σ	0.25 (3.95)	0.26 (4.41)	—	—	—	27.46
Model performance						
OFV	4,290.12	4,265.13	—	—	—	—

RES, relative standard error; CL, clearance; V_1_, central volume; V_2_, peripheral volume; V_3_, peripheral volume; Q_2_, intercompartmental clearance; Q_3_, intercompartmental clearance; OFV, objective function value; BSV, Between-Subject Variability; WSV, Within-Subject Variability; CI, confidence interval; ω(V_1_), ω(V_2_), ω(V_3_), ω(CL), ω(Q_2_), ω(Q_3_): the estimates of between-subject variability of V_1_, V_2_, V_3_,CL, Q_2_, Q_3_, respectively.

Covariate analysis identified body weight (WT) and age as significant covariates affecting CL, and their relationships were described using power functions. The final model equation for clearance estimation is as follows:

$$CL=1.01\times {\left(\frac{WT}{66}\right)}^{0.74}\times {\left(\frac{Age}{72.5}\right)}^{-0.21}$$

This equation demonstrates that clearance increases with increasing body weight and decreases with advancing age, which is consistent with physiological expectations.

Model evaluation and validation results confirmed the reliability and robustness of the final model. The relative standard errors (RSE%) of all structural pharmacokinetic parameters were below 30%, and the RSE% of key parameters (CL、V_2_、V_3_、Q_2_、Q_3_) were all less than 10%, indicating high precision of parameter estimation. Internal validation via 1000-time Bootstrap resampling showed that the median values of Bootstrap-estimated parameters were highly consistent with those of the final model, with narrow 95% confidence intervals (CIs), further supporting the stability of parameter estimation.

Compared with the base model, the OFV of the final model decreased significantly (OFV = −24.99), and this difference was statistically significant, justifying the inclusion of body weight and age as covariates. IIV was described using an exponential model, and its variance (ω) was within the acceptable range. Notably, the IIV of CL decreased after the introduction of covariates, indicating that the model successfully explained this part of population heterogeneity. In addition, the intra-individual residual variability remained stable during model optimization.

### 3.3 Population PD model

A sigmoidal E_max_ model was successfully established to characterise the relationship between ciprofol plasma concentration and its pharmacodynamic effects. The final model parameter estimates, parameter precision, and bootstrap validation results are summarised in Table 3. The effect-site equilibrium half-life (K_e0_) was approximately 1.09 min^-1^, indicating that the distribution equilibrium of ciprofol in the effect site is achieved very rapidly. The baseline BIS value (E_0_) was estimated to be 93.4, which is close to the fully awake state. The maximum effect (Emax) was 45.77, the median effective concentration (EC_50_) was 233.91 ng mL^-1^, and the Hill coefficient (γ) was 3.00.

**TABLE 3:** Population PD parameter estimates of the final PD Model.

Parameters	Estimate (RSE%)	Bootstrap median	95% CI	η-shrinkage (%)	ε-shrinkage (%)
Fixed effects					
K_e0_ (min^-1^)	1.09 (25.20)	1.12	0.72, 1.20	29.72	—
E_0_	93.40 (0.71)	93.41	92.07, 94.66	23.51	—
I_max_	45.77 (3.83)	45.79	42.56, 49.56	26.85	—
IC_50_ (ng·ml^-1^)	233.91 (8.06)	235.62	198.19, 273.67	28.33	—
γ	3.00 (12.92)	3.00	2.35, 3.96	29.23	—
Random effects (BSV)					
ω(IC_50_)	0.09 (35.99)	0.08	0.02, 0.14	—	—
ω(γ)	0.02 (88.18)	0.02	−0.02, 0.15	—	—
Random effects (WSV)					
σ	7.70 (4.81)	—	—	—	29.10
Model performance					
OFV	2,693.11	—	—	—	—

RSE, relative standard error; K_e0_, effect-site equilibration rate constant; E_0_, baseline effect; E_max_, maximum inhibitory effect; EC_50_, plasma concentration producing 50% of E_max_;γ, Hill coefficient; BSV, between-subject variability; WSV, within-subject variability; CI, confidence interval; OFV, objective function value.

Covariate analysis results showed that common physiological covariates such as age, body weight, height, and gender did not significantly improve model fitting. Therefore, no covariates were incorporated into the final PD model. The RSE% of all structural parameters was below 40%, and the RSE% of most key parameters (E_0_, Emax, EC_50_) was less than 10%, indicating good estimation precision. The median values from 1000-time bootstrap validation were highly consistent with the final estimates, further supporting the stability of the model.

### 3.4 Model evaluation

**FIGURE 3:**
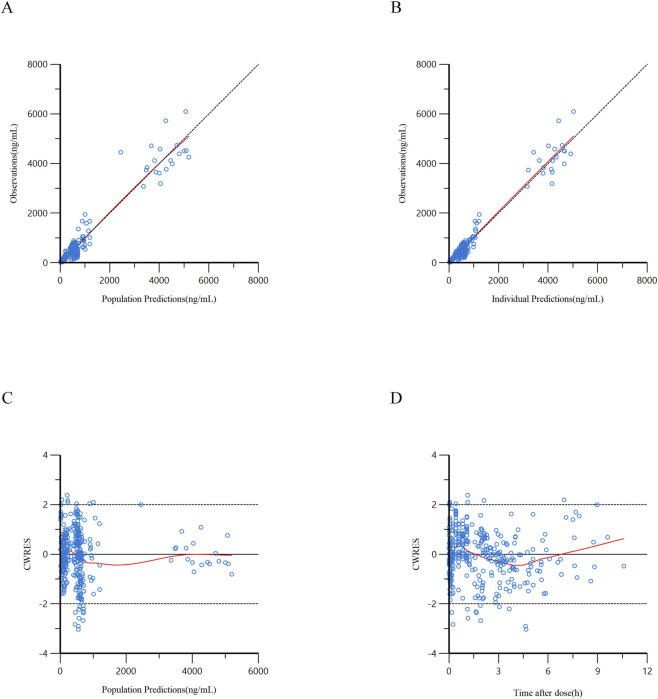
Goodness-of-fit diagnostic plots of the final population PK model of ciprofol. **(A)** Scatter plot of observed concentrations (DV) versus population predicted concentrations (PRED); **(B)** Scatter plot of observed concentrations (DV) versus individual predicted concentrations (IPRED); **(C)** Scatter plot of conditional weighted residuals (CWRES) versus population predicted concentrations (PRED); **(D)** Scatter plot of conditional weighted residuals (CWRES) versus time after drug administration (TAD). In panels **(A,B)**, the black dashed lines represent the y = x reference lines; in panels **(C,D)**, the black solid lines represent the y = 0 reference lines, and the black dashed lines indicate the threshold of │CWRES│ = 2; the red smooth curves reflect the overall trend of the data.

**FIGURE 4:**
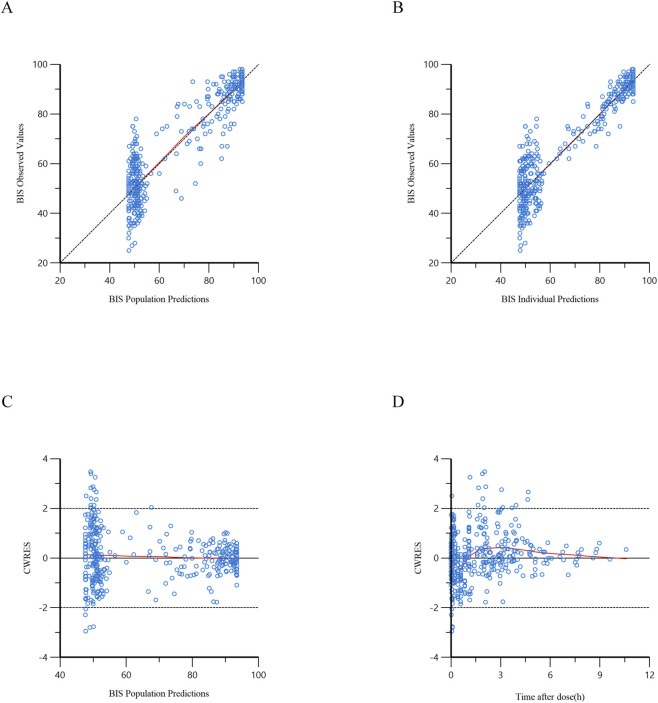
Goodness-of-fit diagnostic plots of the final population PD model of ciprofol. **(A)** Scatter plot of observed BIS values (DV) versus population predicted BIS values (PRED); **(B)** Scatter plot of observed BIS values (DV) versus individual predicted BIS values (IPRED); **(C)** Scatter plot of conditional weighted residuals (CWRES) versus population predicted BIS values (PRED); **(D)** Scatter plot of conditional weighted residuals (CWRES) versus time after drug administration (TAD). In panels **(A,B)**, the black dashed lines represent the y = x reference lines; in panels **(C,D)**, the black solid lines represent the y = 0 reference lines, and the black dashed lines indicate the threshold of │CWRES│ = 2; the red smooth curves reflect the overall trend of the data.

The scatter plots depicting DV against PRED, DV against IPRED, CWRES against PRED, and CWRES against TAD for the final model are illustrated in Figures 3, 4. The results indicated that all data points were randomly distributed around the line of identity (y = x), demonstrating a strong correlation between PRED/IPRED and observed values, as well as adequate model fitting performance. The CWRES versus PRED and CWRES vs. TAD diagnostic plots exhibited a symmetrical distribution of CWRES around the reference line (y = 0), with the majority of values residing within the 2 range. No discernible trends of CWRES with respect to concentration or duration were noted, indicating effective model fitting.

In addition, individual concentration-time profiles (Supplementary Material Figure S1) provided a visual evaluation of the individual predictive ability of the model. The plots showed that the model-predicted concentration trajectories could well capture the overall trends and inter-individual variability of observed concentrations in most patients, further confirming the reliability of the model in describing the complex pharmacokinetic behavior of ciprofol in the elderly population.

**FIGURE 5:**
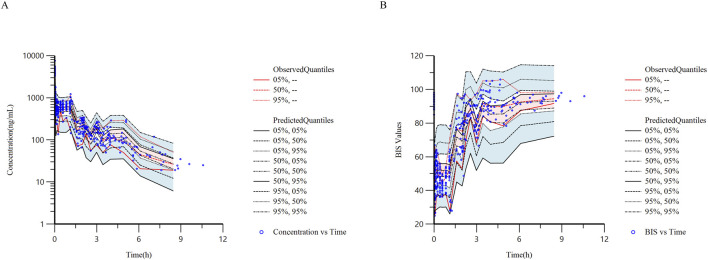
Prediction—corrected visual predictive check (pc-VPC) results of the final ciprofol model. **(A)** Semi-logarithmic pc-VPC plot of the relationship between ciprofol plasma concentration and time; **(B)** pc-VPC plot of the relationship between BIS and time. The black solid lines represent the 5th, 50th, and 95th percentiles of the model-simulated values, respectively; the red solid lines represent the corresponding percentiles of the observed values, respectively; and the scatter points are the actual observed data points.

Figure 5 displays the pc-VPC charts. The median, 5th, and 95th percentiles of the prediction-corrected observed ciprofol concentrations and BIS values significantly coincided with the 95% confidence ranges of the corresponding prediction-corrected predicted values derived from simulated data. The results demonstrated that the developed population PK/PD model can accurately characterize the pharmacokinetic and pharmacodynamic characteristics of ciprofol.

### 3.5 Simulation

Based on the experimental observations, a loading dose of 0.4 mg kg^-1^ administered intravenously over 1 min was first determined to rapidly achieve the target effect of anaesthesia induction. On this basis, simulation analyses of different maintenance dose regimens were performed using the PK/PD model. The optimal regimen was defined as the one that could stably maintain the BIS value within the range of 40–60 for anaesthesia induction and maintenance.

**FIGURE 6:**
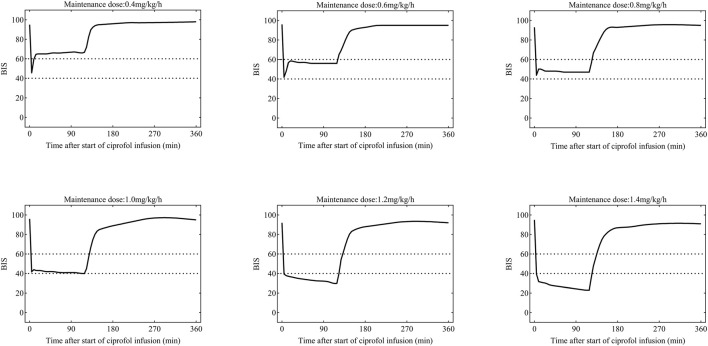
Time-course curves of BIS values under different maintenance infusion rates of ciprofol based on Monte Carlo simulation. Simulation settings: an initial loading dose of 0.4 mg kg^-1^ was infused for 1 min, followed by continuous infusion at rates of 0.4, 0.6, 0.8, 1.0, 1.2, and 1.4 mg kg^-1^·h^-1^ for 2 h, respectively. Each curve reflects the simulated distribution trend of BIS values under the corresponding infusion regimen.

Figure 6 shows the Monte Carlo simulations of BIS changes over time under different administration regimens based on the final PK/PD model. The simulation results indicated that during general anaesthesia in elderly patients, the regimen consisting of a loading dose of 0.4 mg kg^-1^ (infused over 1 min) combined with an initial maintenance dose of 0.6 mg kg·h^-1^, with continuous infusion for 2 h, could sustain the BIS score within the ideal range of 40–60.

## 4 Discussion

This study examined older patients undergoing elective surgery, using ciprofol plasma concentration as the PK index and BIS value as the PD index. The influence of several factors, such as gender, age, height, body mass index (BMI), and laboratory test parameters, on the PK/PD features of ciprofol was carefully examined. A population PK/PD model appropriate for this cohort was developed, intending to furnish a theoretical foundation and model assistance for the personalized administration of ciprofol in clinical practice.

### 4.1 Characteristics of the population PK model

In terms of PopPK, this study confirmed that the three-compartment model could optimally characterize the PK behavior of ciprofol in elderly patients, which is consistent with previously reported model structures (Hu et al., 2021; Liu et al., 2024; Teng et al., 2021). This indicates that the distribution and elimination patterns of ciprofol in the elderly population have not changed fundamentally, but the parameter values exhibit population-specific characteristics. The typical value of CL (1.01 L min^-1^) was slightly higher than that reported in previous studies on the elderly population (Guo et al., 2025). This difference may be attributed to variations in study population characteristics (e.g., comorbidities, baseline liver and kidney function) and surgical types. Moreover, the inclusion of body weight as a significant covariate increased the population typical value of CL. The estimated volume was greater for the deep peripheral compartment (V_3_, 76.79 L) than for the shallow compartment (V_2_, 45.15 L), which may be related to changes in body composition of elderly patients (increased fat proportion and decreased muscle mass). This makes ciprofol, a lipophilic drug, more prone to accumulate in deep tissues such as fat, thus rendering the deep peripheral compartment the main drug reservoir (Thürmann, 2020). This is consistent with the high lipophilicity of ciprofol and its extensive distribution and accumulation in adipose tissues, verifying the physiological rationality of the model structure. The differences in inter-compartmental clearances further reflect the unbalanced transfer rates of the drug between the shallow and deep peripheral compartments, suggesting that clinical attention should be paid to the risk of delayed emergence caused by the slow release of drugs from deep tissues.

Covariate analysis revealed the key sources of inter-individual variability in ciprofol PK among elderly patients. WT and age had significant effects on CL, which is consistent with the general PK rules in the elderly population: increased body weight can enhance clearance capacity, while the physiological decline of liver and kidney function with aging leads to a slight decrease in clearance (Thürmann, 2020). Based on the final model, the influence exponent of body weight on CL was 0.74, which is within the range reported for propofol (Puri, A., Medhi, 2012; Sahinovic et al., 2018; Van Kralingen et al., 2011). Notably, this study found no statistically significant effects of other covariates (including gender, BMI, aspartate aminotransferase, alanine aminotransferase, total bilirubin, total protein, and creatinine clearance) on ciprofol PK. This result is different from some studies on propofol (Araújo et al., 2020; Schüttler and Ihmsen, 2000), which may be due to the masking effect of the general decline in physiological function of the elderly population on inter-individual variability.

### 4.2 PK model and concentration-effect relationship

The relationship between ciprofol plasma concentration and BIS value conforms to the sigmoidal Emax model, which is consistent with literature reports (Hu et al., 2021; Liu et al., 2024; Teng et al., 2021). The key PD parameters highlight the advantages of ciprofol in elderly anesthesia. The K_e0_ was 1.09 min^-1^, indicating that the drug can rapidly reach equilibrium between plasma and the central nervous system, which is consistent with the clinically observed characteristics of rapid onset (1–2 min) and rapid offset of sedation. This parameter is lower than that of propofol (K_e0_: 0.57 min^-1^) (Chen et al., 2024) and superior to that of remimazolam (K_e0_: 1.38 min^-1^) (Lim et al., 2006), making ciprofol suitable for rapid sequence induction and titratable anesthesia. The E_0_ is close to the normal awake level, confirming the validity of the baseline measurement. The Emax indicates that ciprofol can reduce the BIS value by approximately 46 units compared with the baseline, which is sufficient to produce deep sedation/anesthesia without causing excessive suppression. The EC_50_ was 233.91 ng mL^-1^, reflecting the overall sensitivity of this elderly cohort to ciprofol; this value is lower than that reported in young adults (284 ng mL^-1^) (Teng et al., 2021). The γ indicates a relatively steep concentration-effect curve, meaning that minor changes in plasma concentration near EC_50_ can cause significant changes in BIS value. This characteristic facilitates precise titration of anesthesia depth and reduces the risk of inadequate or excessive sedation, which is particularly important for elderly patients with limited physiological reserve.

Covariate analysis showed that factors such as age, body weight, and gender had no significant effects on PD parameters, indicating that the pharmacodynamic responses of elderly surgical patients to ciprofol are relatively homogeneous. This suggests that the inter-individual variability in anesthetic effect is mainly derived from PK factors rather than differences in central nervous system sensitivity.

### 4.3 Model validation and dose simulation

The final PopPK model yielded precise estimates of all pharmacokinetic parameters, which align with the typical properties of GABAA receptor agonists (Schüttler and Ihmsen, 2000). The thorough model validation affirmed the resilience and superior predictive capability of the final PK/PD model. Internal validation with 1,000 bootstrap resampling demonstrated that the median parameter values were remarkably consistent with the final model, and the narrow 95% confidence intervals indicated a high precision of parameter estimation. Goodness-of-fit plots indicated that both individual and population predicted concentrations were closely aligned with the line of identity relative to observed concentrations, and conditional weighted residuals were randomly and symmetrically distributed around zero, devoid of time- or concentration-dependent trends, thereby confirming the absence of systematic bias in the model. The prediction-corrected visual predictive assessment plots further substantiated the model’s predictive capability: the observed median, 5th, and 95th percentiles of ciprofol concentrations and BIS values significantly coincided with the 95% confidence intervals of the simulated data, demonstrating that the model effectively encapsulates the central tendency and inter-individual variability of the data. The PK/PD model simulation indicated that a loading dose of 0.4 mg kg^-1^ (administered over 1 min), followed by an initial maintenance dose of 0.6 mg kg^-1^·h^-1^, can maintain the intraoperative BIS value of older patients within the optimal sedation range of 40–60. Consistent anesthetic depth can be sustained with continuous infusion for over 2 h. This optimal regimen differs from This optimized regimen diverges from the previously recommended starting maintenance dose of 0.8 mg kg·h^-1^ in adult trials (Liu et al., 2024), which primarily considers the diminished clearance and heightened risk of drug buildup in older patients. The maintenance dose was suitably decreased to equilibrate anesthetic efficacy and safety.

The findings from the PK/PD model-based Monte Carlo simulations in this study confirmed that the administration regimen consisting of a loading dose of 0.4 mg kg^-1^ (infused over 1 min) combined with an initial maintenance dose of 0.6 mg kg^-1^·h^-1^can stably maintain the intraoperative BIS values of elderly patients within the optimal target range of 40–60. This regimen is significantly lower than the recommended initial maintenance dose of 0.8 mg kg^-1^·h^-1^ for general adult populations (Liu et al., 2024), reflecting the academic rationale of this study: by fully accounting for the pathophysiological characteristics of elderly patients, such as reduced drug clearance and elevated risk of drug accumulation, an appropriate dose reduction was implemented to balance anesthetic efficacy and safety.

Given the current clinical practice where ciprofol administration for elderly patients largely relies on empirical approaches or passive titration, which is prone to causing delayed emergence or circulatory depression, this study adhered to the principle of “minimum effective dose”. Through the integration of population pharmacokinetic parameters and simulation verification, we confirmed the superiority of the 0.6 mg kg^-1^·h^-1^ infusion rate in achieving the optimal benefit-risk balance, and revealed the critical role of incorporating individual covariates in reducing drug accumulation and accelerating postoperative emergence. This paradigm shift from an experience-driven model to an evidence-based precision medicine model holds profound clinical guiding significance for optimizing the perioperative safety of elderly patients and improving the quality of postoperative recovery.

### 4.4 Analysis of potential drug-drug interactions

In clinical anesthesia practice, ciprofol is frequently administered in combination with a variety of adjunctive drugs, and its potential drug-drug interactions (DDIs) warrant close attention. From a PK perspective, studies have demonstrated that ciprofol is primarily eliminated via UGT1A9-mediated glucuronidation and CYP2B6-mediated oxidation in the liver (Zhou et al., 2021). In contrast, remifentanil undergoes rapid hydrolysis by non-specific plasma and tissue esterases, and cisatracurium is metabolized through Hofmann elimination; neither of these two drugs relies on the conventional hepatic enzyme system. Midazolam, on the other hand, is predominantly metabolized via CYP3A4. Owing to the heterogeneity of the metabolic pathways among these drugs, the likelihood of competitive PK interactions occurring at the clinical doses employed in this study is extremely low. From a PD perspective, ciprofol, remifentanil, and midazolam all exert their effects on the central nervous system, suggesting that significant pharmacodynamic synergism may exist among them. Existing studies have indicated that opioids can significantly reduce the concentration of sedatives required to achieve the desired depth of anesthesia through pharmacodynamic synergistic effects. This phenomenon has been well-documented in the clinical application of propofol. As a novel sedative with a mechanism of action similar to that of propofol, ciprofol may exhibit analogous synergistic effects (Bouillon et al., 2004). In addition, cisatracurium, as a peripheral neuromuscular blocker, does not have direct PD interactions with ciprofol.

To minimize the potential bias of DDIs on the estimation of model parameters, a standardized combined administration regimen was implemented in this study. The aim was to ensure that the derived population parameters could objectively characterize the pharmacological properties of elderly patients under the standard clinical pathway.

### 4.5 Study limitations

Despite its strengths, this study has several limitations that need to be addressed. First, the sample size of this study (*n* = 20) and the single-center study design impose certain limitations on the extrapolation of its conclusions to more diverse surgical populations. Therefore, large-sample, multi-center studies are urgently needed in the future to conduct more extensive external validation of the dosing regimens and model parameters derived from this research. Second, potential drug-drug interactions with concomitant medications (e.g., opioids, benzodiazepines) were not considered in the construction of the ciprofol PK/PD model. In addition, the effects of surgery-related factors (e.g., intraoperative fluid infusion volume, blood transfusion volume, body temperature) or genetic polymorphisms on ciprofol PK/PD were not evaluated. Finally, the PD model was established solely based on BIS values; integrating additional endpoint indicators (e.g., Modified Observer’s Assessment of Alertness/Sedation scale, electroencephalogram spectral entropy) would help improve the comprehensiveness of the model.

## 5 Conclusion

In summary, this study successfully established a population PK/PD model of ciprofol in elderly surgical patients, accurately characterized its pharmacokinetic and pharmacodynamic profiles, clarified the effects of key covariates (WT and age), and optimized the administration regimen through model simulation. This model provides a scientific tool for individualized dose adjustment of ciprofol in elderly patients and also offers methodological reference for conducting larger-scale clinical studies in the future.

## Data Availability

The original contributions presented in the study are included in the article/Supplementary Material, further inquiries can be directed to the corresponding author.
