# Supplementary material for: From pharmacokinetics to precision dosing: optimizing continuous infusion regimens of ciprofol for elderly patients
Source: Front Pharmacol. 2026 Feb 2;17:1764590. doi: 10.3389/fphar.2026.1764590 (PMC12907304; doi:10.3389/fphar.2026.1764590)

**Table S1.** **Comparison of Goodness-of-Fit Metrics for Different Compartment Models.**

| Model Type | OFV | ΔOFV (vs Three-compartment model) | AIC | BIC |
| --- | --- | --- | --- | --- |
| One-compartment model | 4352.67 | 62.55 | 4360.67 | 4372.31 |
| Two-compartment model | 4301.89 | 11.77 | 4313.89 | 4330.25 |
| Three-compartment model | 4290.12 | 0.00 | 4283.13 | 4304.19 |

OFV, objective function value; ΔOFV, Δ objective function value; AIC, Akaike Information Criterion; BIC, Bayesian Information Criterion.

**Table S2.** **Comparison of Goodness-of-Fit Metrics for Different Residual Models.**

| Error Model Type | OFV | RSS | CWRES (mean±SD) |
| --- | --- | --- | --- |
| Proportional Residual Model | 4290.12 | 8962.37 | 0.03±1.02 |
| Additive Residual Model | 4318.56 | 12648.91 | -0.21±1.87 |
| Mixed Residual Model | 4302.69 | 9215.74 | 0.05±1.08 |

OFV, objective function value; RSS, Residual Sum of Squares; CWRES, Conditional Weighted Residuals.

**Table S3.** **Medications and dosage ranges during anesthesia maintenance.**

| Category | Patients (*n*, %) | Dose range |
| --- | --- | --- |
| Maintenance Drugs |  |  |
| Midazolam | 3 (15%) | 0.02-0.05 mg·kg⁻¹ |
| Remifentanil | 20 (20%) | 1.5-4.5 ng·ml⁻¹ |
| Cisatracurium | 16 (80%) | 0.07-0.15 mg·kg⁻¹ |
| Vasoactive Agents |  |  |
| Phenylephrine | 5 (25%) | 0.03-0.1 μg·kg⁻¹·min⁻¹ |
| Ephedrine | 3 (15%) | 5-10 mg |

**Figure S1. Concentration-time profiles of ciprofol in 20 elderly patients**. The solid red lines represent the individual predicted concentrations (IPRED), the dashed black lines represent the population predicted concentrations (PRED), and the circles represent the observed concentrations.


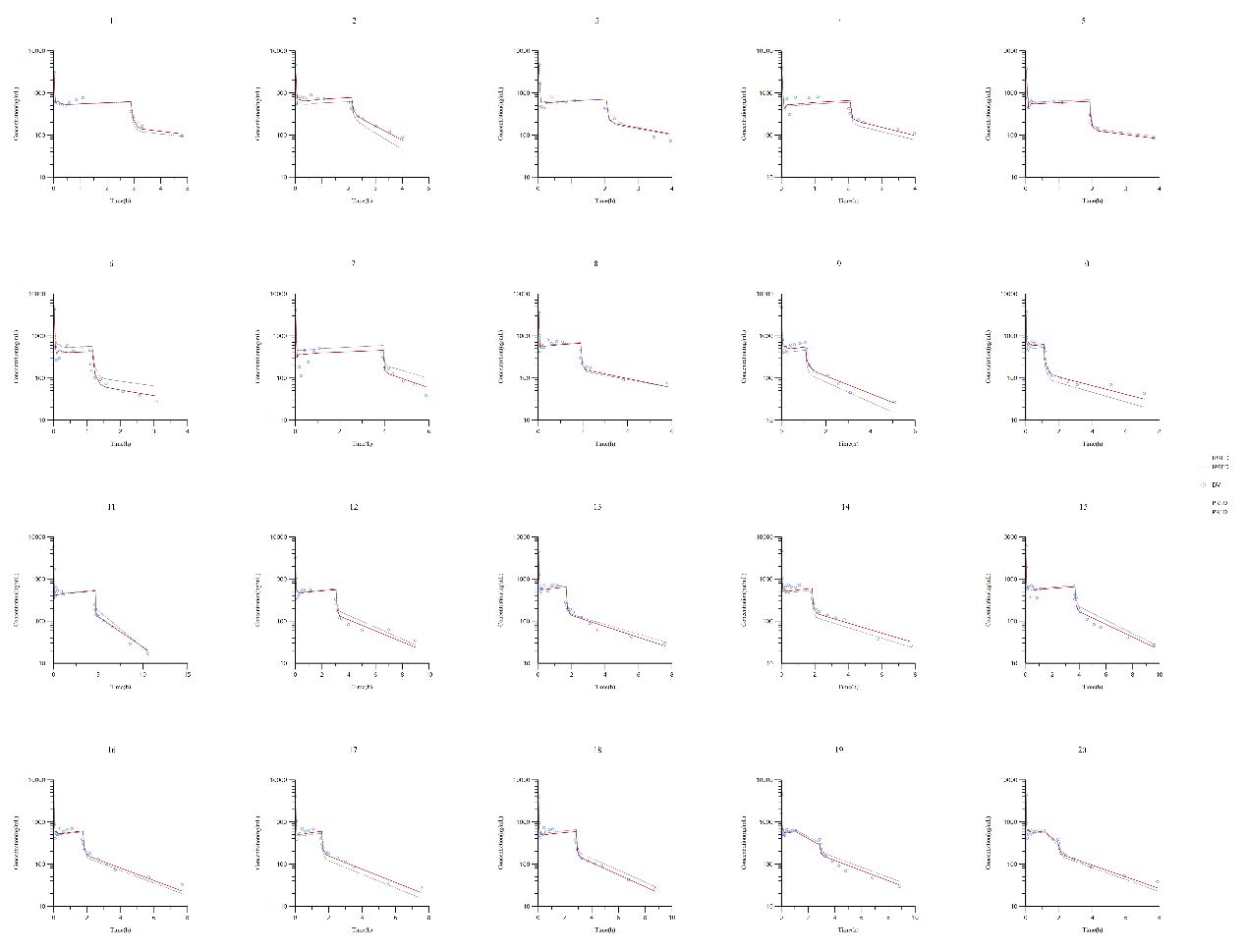


**Figure S2. BIS-time profiles of ciprofol in 20 elderly patients**. The solid red lines represent the individual predicted concentrations (IPRED), the dashed black lines represent the population predicted concentrations (PRED), and the circles represent the observed concentrations.


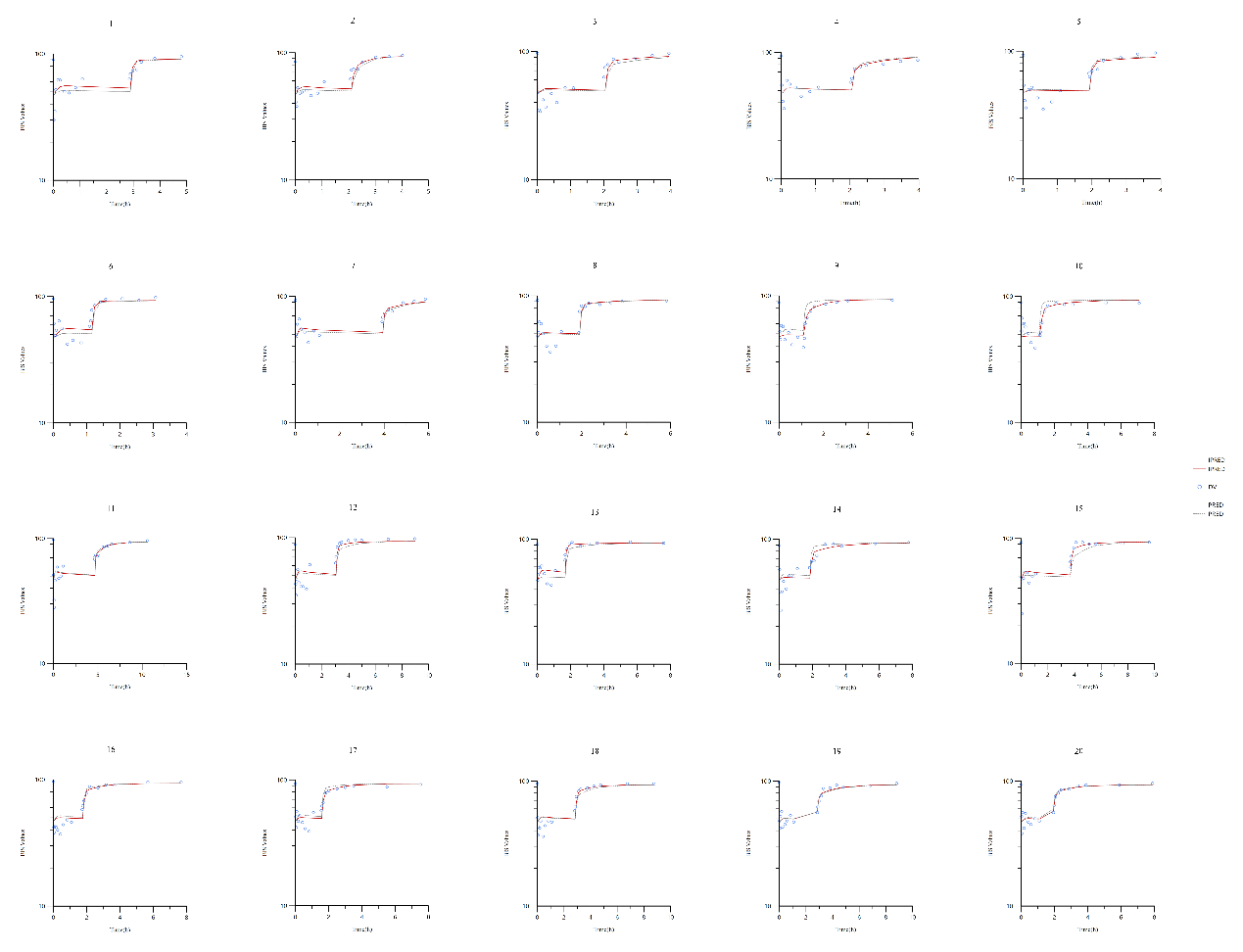

Supplement: Supplementary file 3 [file DataSheet1.docx]
